# Construction and Immunogenicity of a Recombinant Porcine Pseudorabies Virus (PRV) Expressing the Major Neutralizing Epitope Regions of S1 Protein of Variant PEDV

**DOI:** 10.3390/v16101580

**Published:** 2024-10-08

**Authors:** Xian-Qin Jiao, Ying Liu, Xi-Meng Chen, Cheng-Yuan Wang, Jian-Tao Cui, Lan-Lan Zheng, Shi-Jie Ma, Hong-Ying Chen

**Affiliations:** 1 Ministry of Education Key Laboratory for Animal Pathogens and Biosafety, College of Veterinary Medicine, Henan Agricultural University, Zhengdong New District Longzi Lake 15#, Zhengzhou 450046, China; jiaoxianqin@126.com (X.-Q.J.); liuyingeatsunset@126.com (Y.L.); simonarchimonde@126.com (X.-M.C.); w8881119@163.com (C.-Y.W.); cuijiantao1995@163.com (J.-T.C.); lanlan@henau.edu.cn (L.-L.Z.); 2 Animal Health Supervision Institute, Honghu 433200, China

**Keywords:** porcine pseudorabies virus, porcine epidemic diarrhea virus, vaccine

## Abstract

Porcine epidemic diarrhea virus (PEDV) infection causes severe diarrhea and high mortality in neonatal piglets. Pseudorabies causes acute and often fatal infections in young piglets, respiratory disorders in growing pigs, and reproductive failure in sows. In late 2011, pseudorabies virus (PRV) variants occurred in Bartha-K61-vaccine-immunized swine herds, resulting in economic losses to the global pig industry. Therefore, it is essential to develop a safe and effective vaccine against both PEDV and PRV infections. In this study, we constructed a recombinant virus rPRV-PEDV S1 expressing the major neutralizing epitope region (COE, SS2, and SS6) of the PEDV S1 protein by homologous recombination technology and CRISPR/Cas9 gene editing technology, and then evaluated its biological characteristics in vitro and immunogenicity in pigs. The recombinant virus rPRV-PEDV S1 had similar growth kinetics in vitro to the parental rPRV NY-gE^−^/gI^−^/TK^−^ strain, and was proven genetically stable in swine testicle (ST) cells and safe for piglets. PEDV S1-specific antibodies were detected in piglets immunized with rPRV-PEDV S1 on the 7th day post-immunization (dpi), and the antibody level increased rapidly at 14–21 dpi. Moreover, the immunized piglets receiving the recombinant virus exhibited alleviated clinical signs and reduced viral load compared to the unvaccinated group following a virulent PEDV HN2021 strain challenge. Also, piglets immunized with rPRV-PEDV S1 developed a PRV-specific humoral immune response and elicited complete protection against a lethal PRV NY challenge. These data indicate that the recombinant rPRV-PEDV S1 is a promising vaccine candidate strain for the prevention and control of PEDV and PRV infections.

## 1. Introduction

Porcine epidemic diarrhea (PED) is a severe enteric disease of swine caused by porcine epidemic diarrhea virus (PEDV) with 80–100% mortality in neonatal piglets. PEDV was first discovered in Belgium in 1978 [1,2] and subsequently rapidly spread throughout European countries [3,4]. From 1984 to early 2010, PEDV infections occurred in the pig population in China, but there were no large-scale outbreaks [5,6]. However, outbreaks of PED have been seen in the Chinese pig population since the end of 2010, causing enormous economic losses [7,8,9,10,11]. In China, the vaccines used to control PEDV infection at present are conventional inactivated and attenuated vaccines. In 1994, Ma et al. developed an inactivated vaccine based on the cell-adapted CV777 strain [12] and subsequently formulated a bivalent inactivated vaccine for transmissible gastroenteritis virus (TGEV) and PEDV [13]. Tong et al. prepared an attenuated, bivalent TGEV and PEDV vaccine based on the attenuated PEDV vaccine [14]. The inactivated, bivalent TGEV and PEDV vaccine (1999 to present) and the attenuated, bivalent TGEV and PEDV vaccine (2003–2006) are extensively used in the Chinese pig population, and they play important roles in the control of TGEV and PEDV infections [15]. Unfortunately, most current routinely inactivated and attenuated vaccines are not effective or safe enough to control PEDV infection [16]. In addition, attenuated vaccines generally induce long-lasting immunity, with potential reversion and mutation, posing safety issues [17]. The limitations of inactivated vaccines include high manufacturing costs and a lack of long-term immunity [18]. Thus, developing a novel vaccine to control this PED is still the primary task of importance.

PEDV is a single-stranded positive-sense RNA virus belonging to the *Alphacoronavirus* genus within the *Coronaviridae* family. The PEDV genome is approximately 28 kilobases (kb) in length [19] and encodes seventeen non-structural proteins (NSP1–16 and ORF3) and four structural proteins: spike (S), envelope (E), membrane (M), and nucleocapsid (N) proteins. The S gene, known for its high variability among PEDV strains, is used for phylogenetic analysis to determine viral genetic diversity [8,20,21]. The S protein, containing 1383–1386 amino acids (aa) in most strains, is a type-I transmembrane glycoprotein located on the outer surface of the viral particles, and is cleaved at potential N-glycosylation sites into the S1 (aa 1–789) and S2 (aa 790–1383) subunits by foreign or host cell proteases [22]. The capacity of the S protein to bind to host receptors and its role in viral entry determine PEDV invasion and release, host range, tissue tropism, cross-species transmission, and trypsin-dependent proliferation [23]. Importantly, the S protein is the major target for inducing neutralizing antibodies, and six neutralizing epitopes have been identified: the S1^0^ (aa 19–220) [24], S1^A^ (aa 435–485) [25], COE (aa 499–638) [26], SS2 (aa 748–755), SS6 (aa 764–771) [27], and 2C10 (aa 1368–1374) [28]. The core neutralization antigen region of PEDV is the COE region, which has been widely applied to develop PEDV subunit vaccines [16] and has a certain effect. SS2 (748YSNIGVCK755) and SS6 (764LQDGQVKI771) are two core epitopes located on the S protein of PEDV [29]. Accordingly, COE, SS2, and SS6 of PEDV are the primary targets for vaccine development.

Pseudorabies virus (PRV), also known as Aujeszky’s disease (AD), has been prevalent in pig farms worldwide for nearly two hundred years. PRV possesses a double-stranded DNA genome approximately 143 kb in size and is tightly arranged, with at least 70 open reading frames and two inverted repeats, belonging to the subfamily *Alphaherpesvirinae* of the *Herpesviridae* family [30]. PRV has a large genome, and the deletion of one or more genes may reduce its virulence without significantly affecting its growth ability [31] and immunogenicity [32,33]. Several PRV-based vector vaccines have been developed for additional immune protection or enhanced immune effect [34,35,36,37].

Recent studies have increasingly indicated that the use of vaccines based on recombinant PRV live vectors is an emerging and effective strategy for preventing viral infections [38,39,40,41]. In this study, a recombinant virus rPRV-PEDV S1 was constructed to express the major neutralizing epitope region (COE, SS2, and SS6) of the PEDV S1 protein via homologous recombination and CRISPR/Cas9 gene editing technology, and then its biological characteristics in vitro and immunogenicity in pigs were evaluated. This study lays a theoretical and technical foundation for the development of a potential candidate vaccine to prevent and control both PEDV and PRV infections in pigs.

## 2. Materials and Methods

### 2.1. Cells, Viruses, Plasmid

The swine testicle (ST) cell line, PK-15 cell line (ATCC™ CCL-33), green monkey kidney cell line (Vero), and porcine small intestine epithelial cell (IPEC) line were purchased from the China Institute of Veterinary Drug Control, Beijing, China, and cultured in Dulbecco’s modified Eagle medium (DMEM) (HyClone laboratory Inc, Longa, UT, USA) supplemented with 10% fetal bovine serum (FBS) (BI) (37 °C, 5% CO_2_).

The PRV variant NY strain (GenBank accession no. KF130883) was isolated in 2012 from a piglet with neurological symptoms in the Henan province of China, and the variant PEDV HN2021 strain (GenBank accession no. OR707084) was isolated from a diarrhea-infected pig in 2021 [42]. Both strains were stored in our laboratory and used for the virus challenge test of the piglets. The rPRV NY-gE^−^/gI^−^/TK^−^ strain was constructed by deleting virulence factors (TK, gI, and gE) of the PRV variant NY strain [43], on which the recombinant virus strain in this study was constructed. The plasmids involved in the construction process were previously constructed and stored in our laboratory, including the PRV transfer plasmid pG-EGFP [44] and CRISPR/Cas9-EGFP knockout plasmid pX459-gRNA1-EZ-gRNA2 [43]. Mouse monoclonal antibody against PEDV S protein was prepared and preserved by the Animal Biotechnology Center, College of Veterinary Medicine, Henan Agricultural University (Zhengzhou, China).

The experimental procedures were carried out according to the Animal Care and Use Committee of Henan Agriculture University (Approval number SCXK 2013–0001), and performed in accordance with the ‘Guidelines for Experimental Animals’ of the Ministry of Science and Technology (Beijing, China).

### 2.2. Construction of Recombinant Transfer Plasmids

Due to 2 aa deletions occurring in the N-terminal domain of the HN2021 S protein in comparison to CV777 (accession no. AF353511) [42], further alignment with three PEDV S proteins (accession no. AF353511, KP276252, and JN599150) suggested that the major neutralizing epitope regions (COE, SS2, and SS6) of the HN2021 S protein were located at the aa 499–793. A pair of specific primers PEDV S1-F/R was designed and synthesized, containing the restriction enzyme *Bam*H I and the 293 aa epitope region (aa 499–793) (Table 1).

The target S1 gene sequence was first amplified by reverse transcription–polymerase chain reaction (RT-PCR) from viral RNA extracted from the PEDV HN2021 strain, then purified, subsequently cleaved by restriction enzyme *Bam*H I, and finally cloned into digested PRV transfer plasmid pG-EGFP. The constructed recombinant plasmid pG-PEDV S1-EGFP was extracted using the End-free Plasmid Mini Kit II (Omega) according to the manufacturer’s instructions and then verified by restriction enzyme digestion and sequencing.

### 2.3. Construction of Recombinant Virus rPRV-PEDV S1

Firstly, the parental rPRV NY-gE^−^/gI^−^/TK^−^ strain was inoculated into ST cells (80% confluence) in six-well plates, and two hours later, the cells were transfected with the recombinant plasmid pG-PEDV S1-EGFP using transfection reagent Lipofectamine^TM^ 3000 (Invitrogen, Carlsbad, CA, USA) according to the manufacturer’s instructions. When 80% of the cytopathic effects (CPEs) appeared, the transfected cells containing the recombinant virus rPRV-PEDV S1-EGFP were frozen and thawed in a −80 °C refrigerator 3 times. After that, the recombinant virus rPRV-PEDV S1-EGFP was purified by a 96-well plate limited dilution method and a 6-well plate virus plaque purification method. The purified recombinant virus rPRV-PEDV S1-EGFP was identified by PCR amplification and sequencing of the target S1 gene. Finally, the EGFP gene in the genome of the recombinant virus rPRV-PEDV S1-EGFP was knocked out by transfecting CRISPR/Cas9-EGFP knockout plasmid pX459-gRNA1-EZ-gRNA2 into 80% confluent ST monolayers infected with the recombinant virus. The resulting recombinant virus rPRV-PEDV S1 was purified by screening non-green fluorescence plaques as previously described [43], and the absence of the EGFP gene in the genome of the recombinant virus rPRV-PEDV S1 was validated by PCR amplification and sequencing of the incomplete EGFP gene.

### 2.4. Expression of the S1 Protein in the Recombinant Virus

#### 2.4.1. RT-PCR

The recombinant virus rPRV-PEDV S1 was inoculated into ST cells and viral RNA was extracted using the TaKaRa MiniBEST Viral RNA/DNA Extraction Kit Ver.5.0 (Takara, Dalian, China) according to the manufacturer’s instructions. RNA was reverse-transcribed into cDNA using the TIANScriptII RT Kit (TIANGEN, Beijing, China). The expression of the S1 protein in ST cells was measured by PCR amplification using PEDV-specific primers S1-F/R (Table 1).

#### 2.4.2. Western Blot

The recombinant virus rPRV-PEDV S1 was infected with ST cells, which were harvested with RIPA containing 1 mM phenylmethane sulfonyl fluoride (PMSF) (Biyotime, Shanghai, China). After extraction, the S1 protein was separated by 15% sodium dodecyl sulfate–polyacrylamide gel electrophoresis (SDS-PAGE) and transferred onto a nitrocellulose membrane (Solarbio, Beijing, China). The membrane was blocked by 5% skim milk (prepared by PBS). After being washed with PBST (0.5% Tween-20 in PBS), it was incubated with mouse anti-PEDV S antibody (produced by our laboratory, unpublished data) as the primary antibody, and then developed with horseradish peroxidase (HRP)-conjugated goat anti-mouse IgG antibody (Invitrogen Life Technologies, Carlsbad, CA, USA) as the secondary antibody. Finally, imaging with hypersignal detection of protein bands was performed by the West Pico Plus Chemiluminescence Branch (Thermosciences, Waltham, MA, USA) and Chemiluminescence Imaging System (Chemidoc MP Bio-Rad, Hercules, CA, USA).

### 2.5. Biological Characteristics of Recombinant Virus

#### 2.5.1. Viral Growth in Different Cell Types

To determine the in vitro growths of recombinant virus rPRV-PEDV S1 in different cell types, it was serially diluted 10-fold with DMEM, and then inoculated into ST, PK-15, Vero, and IPEC cells with 100 μL per well in 96-well plates. The plates were examined daily for a discernible CPE, and the final reading was made after 7 days. Control wells containing uninoculated cell cultures were included in the test. The tissue culture infectious dose 50% assay (TCID_50_) was determined in ST, PK-15, Vero, and IPEC cells, and calculated according to the Reed and Muench method [45].

#### 2.5.2. Growth Kinetics

In order to analyze the growth characteristics of the recombinant virus rPRV-PEDV S1, rPRV-PEDV S1 and parental strain rPRV NY-gE^−^/gI^−^/TK^−^ were cultured in 12-well plates at a multiplicity of infection (MOI) of 1 per cell. Two h after inoculation, the cells were washed three times with PBS (0.1 mol/L) and then overlaid with DMEM (2% FBS). The infected cells and supernatants were harvested at 0, 4, 8, 12, 16, 20, 24, 28, 32, and 36 hpi for titration. All samples were taken at each time point in triplicate, and the results are presented as the mean ± standard deviation (SD) of the triplicate assays. The TCID_50_ of each time point sample was determined, and a one-step growth curve of the virus was plotted.

#### 2.5.3. Determination of Physicochemical Properties

The procedures to evaluate the physicochemical properties of the recombinant virus rPRV-PEDV S1 were performed as described previously [37]. Briefly, the virus was divided into different groups and subjected to different treatments, including exposure to UV irradiation for 30 min, treatment with 0.15% formaldehyde for 24 h, exposure to pH conditions of 3.0 or 11.0 for 1 h followed by adjustment to pH 7.0, treatment with 4.8% chloroform for 30 min, and incubation in 20% ether at 4 °C for 24 h. The parental strain rPRV NY-gE^−^/gI^−^/TK^−^ was also treated in the same way. Subsequently, 100 μL of each treated sample was inoculated into ST cells, and the CPE was observed daily. Recombinant virus without any treatment was used as a positive CPE control.

### 2.6. Genetic Stability

To determine the genetic stability of the PEDV S1 gene introduced into the recombinant virus rPRV-PEDV S1, the recombinant virus was serially passaged 20 times in ST cells. The insertion of the PEDV S1 gene was verified by PCR using PEDV-specific primers S1-F/R (Table 1) in the 5th, 10th, 15th, and 20th generations. The parental rPRV NY-gE^−^/gI^−^/TK^−^ was used as a control. Subsequently, PCR products were sequenced and aligned to evaluate the genetic stability.

### 2.7. Safety Test and Immunogenicity of Recombinant Virus in Piglets

To test the safety and immunogenicity of the recombinant virus rPRV-PEDV S1, eighteen two-week-old healthy piglets were purchased, all of which were confirmed negative for PRV, PEDV, TGEV, porcine delta coronavirus, and porcine rotaviruses by virus-specific PCR or RT-PCR of rectal swabs. The piglets were randomly divided into two sets (N = 9), each of which was further divided into three groups in accordance with the principle of randomized block design. Piglets in each group (n = 3) were reared in separate rooms and provided with sterilized feed and water. In Set 1, piglets in each group received a subcutaneous injection (SC) with 2 mL of rPRV-PEDV S1 /NA (10^6.0^ TCID_50_), 2 mL of the commercial PEDV-TGEV inactivated vaccine (Wuhan Keqian Biology Co., Ltd., Wuhan, China), or 2 mL of DMEM, respectively. Similarly, in Set 2, piglets in each group received SC with 2 mL of rPRV-PEDV S1, 2 mL of the commercial Bartha-K61 attenuated vaccine (Harbin Pharmaceutical Group Bio-vaccine Co., Ltd., Harbin, China), or 2 mL of DMEM, respectively.

During the period of the experiment, clinical manifestations were monitored daily and rectal temperature was measured every 3 days to evaluate the safety of the recombinant virus in piglets. Blood samples were collected weekly from the ear vein at weeks 1, 2, 3, and 4 after the initial immunization. The sera were isolated and stored at −80 °C.

#### 2.7.1. Enzyme-Linked Immunosorbent Assay (ELISA)

According to the instructions of the reagent, PEDV S-specific antibodies in the serum samples from Group 1 were identified using the PEDV S Antibody Test Kit (Biovet, Quebec, Canada), and PRV gB-specific antibodies in the serum samples from Group 2 were identified using the PRV gB Antibody Test Kit (IDEXX Laboratories, Inc., Westbrook, ME, USA).

#### 2.7.2. Neutralizing Antibody Assay

The collected sera were inactivated at 56 °C for 30 min and then continuously double-diluted (1:2n) with DMEM. Subsequently, the diluted sera were mixed with 200 TCID_50_ of virus solution and incubated at 37 °C with 5% CO_2_ for 1 h. The mixture was added into ST cell monolayers in 96-well plates, and CPE was recorded for 5–7 days. The average neutralizing antibody titer of three measurements was calculated according to the Reed–Muench method.

### 2.8. Challenge Experiments

Four weeks after the first immunization, all piglets in Set 1 were challenged by oral administration of 4 mL (10^4.25^ TCID_50_/mL) of the PEDV HN2021 strain, while all piglets in Set 2 were challenged by SC of 1 mL (10^4.0^ TCID_50_/mL) of the PRV variant NY strain. After the challenge, clinical signs were monitored daily, including mental state, temperature change, feed intake, vomiting, diarrhea, cough, and death. All piglets in Set 1 were euthanized after being challenged with the PEDV HN2021 strain for two weeks; in these piglets, PEDV loads were detected by RT-qPCR [46] in the intestines and feces. All piglets in Set 2 were euthanized after being challenged with the PRV variant NY strain for two weeks; in these piglets, PRV loads were detected by qPCR [47] in various tissues, including the heart, liver, spleen, lung, kidney, brain, intestine, and lymph nodes. Total protection was defined as the absence of detectable virus.

### 2.9. Statistical Analysis

All experimental data were presented as mean ± standard error (SE) by triplicates. Differences between groups were analyzed by two-tailed Student’s t-test and/or one-way analysis of variance (ANOVA) using GraphPad PrismTM 8.0 (GraphPad Software Inc., La Jolla, CA, USA). Values of *p* ≤ 0.05 were considered statistically significant (* *p* < 0.05; ** *p* < 0.01; *** *p* < 0.005; and **** *p* < 0.001), and no significant difference was indicated by “ns” (*p* > 0.05).

## 3. Results

### 3.1. Construction of Recombinant Pseudorabies Virus

**Figure 1:**
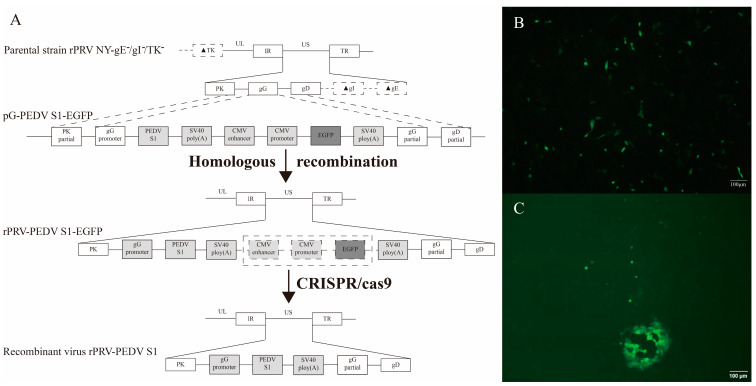
Construction of the recombinant virus rPRV-PEDV S1. (**A**) Construction flowchart. (**B**) The green fluorescent shown in ST cells after transfection with rPRV-PEDV S1-EGFP. (**C**) The plaque of rPRV-PEDV S1-EGFP observed for the first time. Black solid triangles (▲) represent the deletion of the gene.

**Figure 2:**
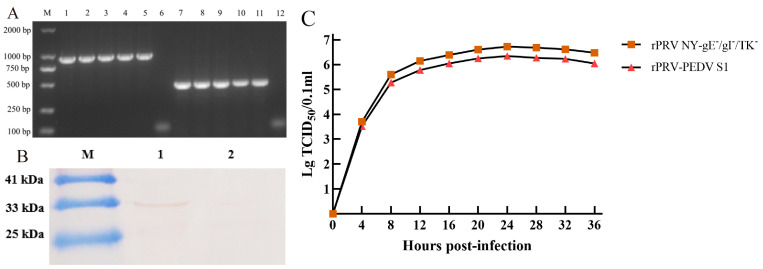
Identification of the recombinant virus rPRV-PEDV S1. (**A**) The amplification results of S1 and EGFP genes using the DNA of the two recombinant viruses. M: DL Marker 2000; lane 1–5: the PEDV S1 gene amplified from the purified rPRV-PEDV S1-EGFP; lane 7–11: shortened EGFP fragments amplified from the purified rPRV-PEDV S1 following CRISPR/Cas9-mediated knockdown of the EGFP gene; lane 6 and 12: negative control. (**B**) Western blot analysis of the expression of the PEDV S1 protein. lane 1: S1 protein expressed in ST cells infected with rPRV-PEDV S1; lane 2: the parental rPRV NY-gE^−^/gI^−^/TK^−^ strain control. (**C**) One-step growth curve of rPRV-PEDV S1 (▲) and rPRV NY-gE^−^/gI^−^/TK^−^ (■) in ST.

As was depicted in Figure 1A, recombinant virus rPRV-PEDV S1 was constructed. Recombinant virus rPRV-PEDV S1-EGFP was generated by transfecting the recombinant plasmid pG-PEDV S1-EGFP into ST cells infected with parental rPRV NY-gE^−^/gI^−^/TK^−^. Under the inverted microscope, the green fluorescent (Figure 1B) in ST cells was observed after transfection, and the plaque of rPRV-PEDV S1-EGFP was obtained for the first time (Figure 1C). After six rounds of plaque purification, the presence of the PEDV S1 gene in the viral genome was confirmed by PCR, with a predicted DNA band (897 bp) detected (Figure 2A), which indicated successful construction of the recombinant virus rPRV-PEDV S1-EGFP.

Subsequently, the purified rPRV-PEDV S1-EGFP was co-transfected with plasmid PX459-gRNA1-EZ-gRNA2 into ST cells to rescue recombinant virus rPRV-PEDV S1. rPRV-PEDV S1 was purified by screening the non-green-fluorescent plaques for three rounds. Finally, the amplified EGFP fragment was found to be 436 bp, which was shorter than expected (1668 bp), through PCR using the EGFP-specific primers EGFP-F/R (Figure 2A). A 1232 bp deletion in the EGFP expression cassette gene of the rPRV-PEDV S1 genome was confirmed by sequencing. These data revealed that the PEDV S1 gene was successfully inserted into the PRV genome via homologous recombination, and that the EGFP expression cassette gene was deleted via the CRISPR/Cas9 system.

### 3.2. Expression of PEDV S1 Protein in Recombinant Virus

To verify the expression of the PEDV S1 protein in ST cells, the PEDV S1 gene was amplified by RT-PCR, with a predicted fragment (897 bp). The sequencing results showed that the nucleotide sequence of the PEDV S1 gene was identical to S1 (OR707084).

Furthermore, the expression of the PEDV S1 protein in the recombinant virus rPRV-PEDV S1 was corroborated by Western blot. It was found that the specific band with a weight of 32.2 kDa was detected in ST cells infected with rPRV-PEDV S1, which was recognized by the anti-PEDV S primary antibody (Figure 2B, lane 1). However, no protein band was observed in ST cells infected with parental rPRV NY-gE^−^/gI^−^/TK^−^ (Figure 2B, lane 2). These results showed that the recombinant virus rPRV-PEDV S1 was successfully constructed.

### 3.3. Biological Characteristics of Recombinant Virus rPRV-PEDV S1

In vitro growths of the recombinant virus rPRV-PEDV S1 were determined by TCID_50_/mL in different cell types. The TCID_50_ of the recombinant virus on ST, Vero, PK-15, and IPEC cells was 6.325 log10/mL, 6.125 log10/mL, 5.5 log10/mL, and 5.375 log10/mL, respectively, whereas the parental strain rPRV NY-gE^−^/gI^−^/TK^−^ had measurements of 6.775 log10/mL, 6.025 log10/mL, 5.65 log10/mL, and 5.15 log10/mL for ST, Vero, PK-15, and IPEC cells, respectively.

The results showed that the optimal proliferating cell type for the recombinant virus rPRV-PEDV S1 was ST cells, followed by Vero cells and PK-15 cells, while the sensitivity on IPEC cells was the worst. The TCID_50_ of the recombinant virus rPRV-PEDV S1 in this study was slightly lower than that of the parental strain, which may be due to continuous passage enabling the virus to adapt to cell proliferation. Furthermore, the genetic characteristics of the recombinant virus were stable. The results indicated that ST cells were more suitable for the proliferation culture of the recombinant virus rPRV-PEDV S1.

The in vitro single-step growth curves (Figure 2C) showed that the growth dynamics of rPRV-PEDV S1 were similar to those of rPRV NY-gE^−^/gI^−^/TK^−^. Specifically, although the virus titer of the recombinant strain was high (>6 log10/mL TCID_50_), it was lower than that of the parent strain. There is no significant difference in viral titer between the two viruses (*p* > 0.05), which indicates that the insertion of the exogenous gene did not affect the proliferation of the viral vector.

The results of the evaluation of physicochemical characteristics showed that the recombinant virus rPRV-PEDV S1 and its parent strain rPRV NY-gE^−^/gI^−^/TK^−^ were sensitive to 0.15% formaldehyde, high temperature at 60 °C, pH adjusted to 3.0 or 11.0, and 20% ether. In contrast, rPRV-PEDV S1 and rPRV NY-gE^−^/gI^−^/TK^−^ maintained infectivity, with 10^2.875^ TCID_50_/mL and 10^2.215^ TCID_50_/mL, respectively, after 4.8% chloroform treatment, and rPRV-PEDV S1 and rPRV NY-gE^−^/gI^−^/TK^−^ were highly infectious after 30 min UV irradiation, with 10^5.875^ TCID_50_/mL and 10^5.625^ TCID_50_/mL, respectively. The results showed the recombinant virus was sensitive to pH and chemical reagents, but it was stable under UV irradiation, which was consistent with the general physicochemical characteristics of PRV.

### 3.4. Genetic Stability and Safety Test of Recombinant Virus in Piglets

After the rPRV-PEDV S1 was consecutively passaged for 20 generations in ST cells, the 897bp-fragment of the PEDV S1 gene was amplified from viral DNA extracted from the 5th, 10th, 15th, and 20th generations of recombinant virus rPRV-PEDV S1; the sequencing results of the S1 gene were completely consistent with the S1 gene sequence of recombinant plasmid pG-PEDV S1-EGFP, and no mutation occurred, confirming that the generated recombinant virus rPRV-PEDV S1 carrying the PEDV S1 gene was genetically stable.

After being immunized with recombinant virus rPRV-PEDV S1, the piglets exhibited normal mental state, feeding behavior, and rectal temperatures, and there were no deaths among them, which showed that the recombinant virus was safe for piglets.

### 3.5. Specific Antibody Response Induced by the Recombinant Virus in Piglets

**Figure 3:**
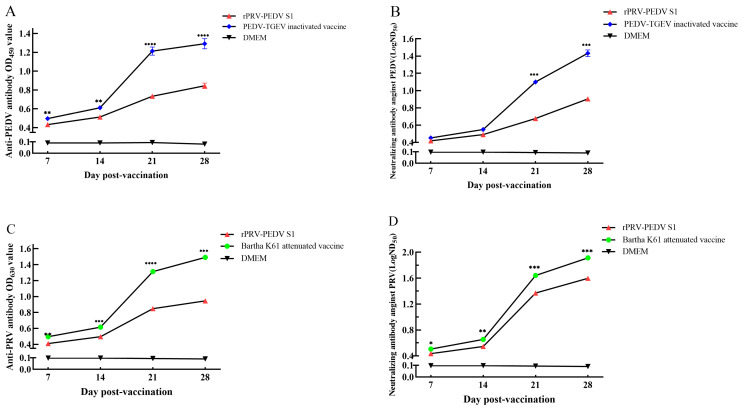
The detection of viral antibodies. (**A**) The S-specific antibodies were calculated by ELISA. (**B**) Detection results of anti-PEDV-neutralizing antibody titer in the serum of immunized piglets. (**C**) The gB-specific antibodies were calculated by ELISA. (**D**) Detection results of anti-PRV-neutralizing antibody titer in the serum of immunized piglets. There was a statistical difference in the labeling of the rPRV-PEDV S1 group (▲) and the PEDV-TGEV inactivated vaccine group (♦) in (**A**,**B**), and the rPRV-PEDV S1 group (▲) and the Bartha-K61 attenuated vaccine group (●) in (**C**,**D**). * *p* < 0.05; ** *p* < 0.01; *** *p* < 0.005; and **** *p* < 0.001.

In Set 1, the detection results of the anti-PEDV S antibody (PEDV-S-Abs) from ELISA and neutralizing antibody assays indicated that the rPRV-PEDV S1 group, as well as the PEDV-TGEV inactivated vaccine group, exhibited significant antibody production at 7 days post-immunization (dpi) (Figure 3A,B). Subsequently, PEDV-S-Abs in both the rPRV-PEDV S1 group and PEDV-TGEV inactivated vaccine group demonstrated a rapid increase between 14 and 21 dpi, followed by a gradual rise from 21 to 28 dpi. Notably, ELISA results for PEDV-S-Abs between the rPRV-PEDV S1 group and the PEDV-TGEV inactivated vaccine group showed significant differences at 7 dpi (*p* < 0.01) (Figure 3A), while neutralizing antibody assays between them revealed significant differences at 21 dpi (*p* < 0.005) (Figure 3B). In brief, the trends of PEDV-S-Abs in both groups were consistent across ELISA and neutralizing antibody detection. These findings suggest that the overall level of PEDV-S-Abs in the PEDV-TGEV inactivated vaccine group was superior to that observed in the rPRV-PEDV S1 group. In contrast, no detectable levels of PEDV-S-Abs were observed in the DMEM control group (DMEM group) over a four-week period.

In Set 2, the detection results of the anti-PRV gB antibody (PRV-gB-Abs) from ELISA and neutralizing antibody assays show a similar result to those in Set 1, indicating that the overall levels of PRV-gB-Abs in the rPRV-PEDV S1 group were lower than those in the Bartha-K61 attenuated vaccine group (Figure 3C,D). Specifically, PRV-gB-Abs was detected as positive at 7 dpi by both ELISA and neutralizing antibody assays in both the rPRV-PEDV S1 group and the Bartha-K61 attenuated vaccine group. However, the PRV-gB-Abs in the rPRV-PEDV S1 group demonstrated superior performance in neutralizing antibody assays compared to ELISA, aligning more closely with that observed in the Bartha-K61 attenuated vaccine group (*p* < 0.005). These findings suggest that rPRV-PEDV S1 may exhibit comparable efficacy to the Bartha-K61 attenuated vaccine.

The above results suggest that the recombinant virus can induce specific antibody responses against PRV and PEDV in pigs.

### 3.6. Protection of Piglets against the Virulent Challenge of PEDV

At four weeks after the first immunization, all piglets in Set 1 were challenged by oral administration of 4 mL (10^4.25^ TCID_50_/mL) PEDV HN2021 strain. Two out of the three piglets from the rPRV-PEDV S1 group (n = 3) exhibited mild diarrhea within 36 h after challenge, followed by subsequent recovery. The third piglet from the rPRV-PEDV S1 group and all three piglets from the PEDV-TGEV inactivated vaccine group (n = 3) displayed normal appetite and good mental state. Nevertheless, two piglets in the DMEM group developed diarrhea, lethargy, and decreased appetite within 24 h of exposure to PEDV variant HN2021, followed by the remaining piglet experiencing lethargy and decreased appetite. One piglet in the DMEM group progressed to severe clinical symptoms (e.g., watery diarrhea, dehydration, and vomiting) on the third day after challenge and eventually died on the fifth day, whereas the other two piglets gradually recovered at 14 d post-challenge.

**Figure 4:**
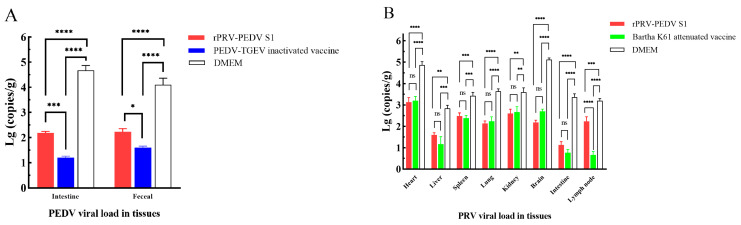
Quantification of viral loads in tissue samples of piglets against virulent challenge. (**A**) Of PEDV, (**B**) Of PRV. * *p* < 0.05; ** *p* < 0.01; *** *p* < 0.005; and **** *p* < 0.001.

After euthanasia, the intestines and feces were collected for the detection of PEDV loads. The RT-qPCR results revealed that the PEDV loads in the intestine were higher than those in the feces. In more detail, the PEDV loads in the intestines and feces of piglets in the rPRV-PEDV S1 group were significantly lower than those in the DMEM group (*p* < 0.001) but higher than those immunized with the commercial PEDV-TGEV inactivated vaccine (*p* < 0.05) (Figure 4A). These results demonstrate that following PEDV challenge, the immunized piglets receiving the recombinant virus exhibited alleviated clinical signs and reduced viral loads compared to the unvaccinated group. However, its effectiveness was diminished relative to the PEDV-TGEV inactivated vaccine.

### 3.7. Protection of Piglets against the Virulent Challenge of PRV

For Set 2, no clinical symptoms were observed in piglets from the rPRV-PEDV S1 group (n = 3) within 2 weeks of challenge by the PRV variant NY strain. At 120 h post-challenge, one piglet in the Bartha-K61 attenuated vaccine group (n = 3) exhibited initial neurological symptoms but subsequently recovered, while the other two piglets remained healthy during the 2-week period. Following challenge with the PRV variant NY strain for 96 h, two out of three piglets in the DMEM group (n = 3) initially displayed neurological symptoms, diarrhea, and vomiting, and eventually succumbed to the disease; meanwhile, the remaining piglet developed symptoms later but gradually recuperated.

Various tissues were collected for the detection of PRV loads after euthanasia. The qPCR results demonstrated varying PRV loads across different organs, with the highest levels being observed in the heart and brain, and the lowest levels found in the small intestine and lymph nodes (Figure 4B). Viral loads in various tissues of vaccinated piglets from both groups were all lower than those in the DMEM group (*p* < 0.01). With the exception of the lymph nodes (*p* < 0.001), no significant differences were observed between the rPRV-PEDV S1 group and the Bartha-K61 attenuated vaccine group (*p* > 0.05). These data confirm that recombinant virus rPRV-PEDV S1 exhibits a favorable protective effect compared with Bartha-K61 attenuated vaccine.

## 4. Discussion

In 2013, a highly pathogenic PEDV outbreak emerged in the United States [48,49] and subsequently quickly spread throughout the pig industry in Europe, North America, and Asia, posing a serious economic threat to the pig industry worldwide [3,20,50,51,52]. To manage PEDV outbreak, conventional inactivated and attenuated vaccines are commonly used for immunization. Live attenuated vaccines (LAVs) are renowned for their high immunogenicity and often require only a single dose to establish protective immunity, but the development of PEDV LAVs is constrained by the risk of reversion to their virulent wild-type forms [53,54]. Inactivated vaccines are known for their exceptional safety and straightforward production process, albeit the immune response triggered by inactivated vaccines is less comprehensive and less enduring than that induced by LAVs, leading to the need for multiple doses for effective immunization. Moreover, research has been conducted on novel vaccines, such as subunit, nucleic acid, viral vector, and virus-like particle (VLP) vaccines [55]. Li et al. reported that a flagellin-adjuvanted PED subunit vaccine rSF-COE-3D could provide better protection against the challenge of a highly pathogenic PEDV variant [56]; however, subunit vaccines are expensive, have low immunogenicity, and require adjuvants. VLP vaccine with a B-cell epitope from PEDV incorporated into hepatitis B virus core capsid provides clinical alleviation against PEDV in neonatal piglets through lactogenic immunity [57]. Nevertheless, because of the high production costs and low yield, the application of VLP vaccines is limited. Nucleic acid vaccines, such as DNA and mRNA vaccines, offer several advantages: they are cost-effective and safe, can be rapidly produced, and are relatively straightforward to design [58,59]. Bivalent DNA vaccines combining PEDV with TGEV have been created but not yet tested in clinical trials due to limited immunogenicity [60,61]. The lipid nanoparticle (LNP)-encapsulated mRNA (mRNA-LNP) vaccine, encoding the full-length PEDV S protein [62] that induced robust PEDV-specific humoral and cellular immune responses in vivo, and protected actively immunized piglets against PEDV, is under development.

It has been reported that attenuated PRV with the deletion of TK and gE genes can be used as a vector to express foreign proteins [63,64], which can infect host cells after immunization; the genome of PRV can express foreign proteins in cells, and then the foreign proteins are secreted out of cells to induce humoral immunity and produce antibodies against foreign proteins. Consequently, attenuated PRV may be a promising candidate vaccine vector for the expression and delivery of other antigens to confer protection against both PR and other viral diseases [64,65,66]. These studies have shown that recombinant viral vectors are a promising vaccine platform, combining the advantages of live attenuated vaccines and subunit vaccines and avoiding some of the shortcomings of each. They are relatively safe and inexpensive and are being actively developed. Various methods for expressing the PEDV S1 region or regions containing neutral epitopes have demonstrated good immunogenicity [67,68]. After pregnant sows were vaccinated with the PEDV S1 protein, passive immunity could be transmitted to unweaned piglets via the colostrum, helping the piglets resist PEDV infection [68]. Since 2011, farms immunized with Bartha-K61 have experienced PR outbreaks caused by PRV variants. Therefore, researchers tried to generate several gene mutant vaccines involving gE/TK, gI/gE, and gI/gE/TK deletion based on different PRV variants [63,69]. These vaccines provided adequate protection against the PRV variant challenge. A gE/gI/TK-deleted recombinant virus rPRV NY-gE^−^/gI^−^/TK^−^ strain was constructed in our laboratory based on the PRV variant NY strain, which could provide complete protection against the lethal PRV NY strain challenge in mice. Hence, rPRV-PEDV S1 containing the major neutralizing epitope region (COE, SS2, and SS6) of PEDV S1 was constructed based on the rPRV NY-gE^−^/gI^−^/TK^−^ strain via homologous recombination and CRISPR/Cas9 gene editing technology to enhance the immunogenicity of the S1 protein of PEDV and assess its efficacy for PEDV and PRV.

In vitro, the biological characteristics of recombinant virus rPRV-PEDV S1 were similar to those of parental rPRV NY-gE^−^/gI^−^/TK^−^, including culture characteristics, growth kinetics, and physicochemical characteristics. Moreover, the genetic stability of rPRV-PEDV S1 indicated that the insertion of the exogenous PEDV S1 gene did not affect the normal replication of parental rPRV NY-gE^−^/gI^−^/TK^−^. The safety test of the recombinant virus demonstrated its non-lethality in piglets, as those inoculated with the rPRV-PEDV S1 recombinant virus did not succumb. To assess the immunogenicity and protective efficacy of the recombinant virus against PEDV and PRV, specific antibody responses to PRV or PEDV were evaluated through neutralizing antibody assays and ELISA. Additionally, viral loads in piglets following PEDV or PRV challenge were measured. These results demonstrated that rPRV-PEDV S1 could induce specific immune responses against PRV and PEDV. For PEDV, the level of PEDV-S-Abs was consistent with the antibody level corresponding to the PEDV loads in RT-qPCR, indicating that rPRV-PEDV S1 immunization could induce a certain level of PEDV antibodies, yet the immune effect of rPRV-PEDV S1 was not as good as that of the commercial PEDV-TGEV inactivated vaccine. For PRV, the level of PRV-gB-Abs in the rPRV-PEDV S1 group was more closely aligned with that observed in the Bartha-K61 attenuated vaccine group (*p* < 0.005), and quantification of viral loads in tissue samples revealed that no significant differences were observed between the rPRV-PEDV S1 group and the Bartha-K61 attenuated vaccine group (*p* > 0.05), except for in the lymph nodes (*p* < 0.001), suggesting that the immune response elicited by rPRV-PEDV S1 is comparable to that induced by the Bartha-K61 attenuated vaccine. The aforementioned results demonstrated that the immunogenicity of recombinant virus rPRV-PEDV S1 was better against PRV and less effective against PEDV compared with the commercial vaccine. These results demonstrate that the recombinant virus alleviated clinical signs and lowered the viral load in comparison to the unvaccinated group in actively immunized piglets after PEDV challenge. A good candidate vaccine not only requires good biological safety but also a good immune protection effect [70]. Taken together, these data suggest the recombinant rPRV-PEDV S1 is safe and effective for piglets, and is a promising vaccine candidate strain for the prevention and control of PEDV and PRV variant infection.

In summary, recombinant virus rPRV-PEDV S1 was generated by homologous recombination and the CRISPR/Cas9 system, and further evaluated for its biological characteristics in vitro and immunogenicity in pigs, which indicated that rPRV-PEDV S1 was a promising multivalent genetic engineering vaccine candidate to control PEDV and PRV variant infection.

## Figures and Tables

**Table 1:** List of primer sequences used in this study.

Primer Name	Nucleotide Sequence (5′-3′)	Product Size
S1-F	GAGGATCC**CT**ATTTCTTTTGTTACTCTGC	897
S1-R	GCGGATCCACTCATACTAAAGTT	
EGFP-F	GATTCTGTGGATAACCGTAT	1668
EGFP-R	ACAATTTACGCCTTAAGAT	

Note: The bolded **CT** is inserted as an extra codon to make the codon number a multiple of 3, without affecting the gene’s transcription or translation.

## Data Availability

The data that support the findings of this study are openly available in this manuscript.
